# Humping Formation and Suppression in High-Speed Laser Welding

**DOI:** 10.3390/ma15072420

**Published:** 2022-03-25

**Authors:** Boce Xue, Baohua Chang, Shenghua Wang, Runshi Hou, Peng Wen, Dong Du

**Affiliations:** 1 Department of Mechanical Engineering, Tsinghua University, Beijing 100084, China; xbc17@mails.tsinghua.edu.cn (B.X.); bhchang@tsinghua.edu.cn (B.C.); 2 Hangzhou Kaierda Robot Technology Co., Ltd., Hangzhou 311232, China; wangsh@kaierda.cn (S.W.); hours@kaierda.cn (R.H.); 3 Key Laboratory for Advanced Materials Processing Technology, Ministry of Education, Beijing 100084, China

**Keywords:** high-speed laser welding, humping, numerical simulation, high-speed imaging, TIG arc

## Abstract

Increasing welding speed can promote the productivity of laser welding. However, humping defects often occur, which limits the application of this strategy. The existing explanations for the humping formation remain vague, and mitigation and suppression methods are limited. In this research, high-speed imaging experiments and numerical simulation of the high-speed laser welding process are performed. Through careful examination, the humping phenomenon is explained. At high welding speed, the high-speed melt flow caused by recoil pressure is hindered by the solidified region in the melt pool, leading to the occurrence of a swelling. The swelling then grows, forming a valley in front of the swelling under the effect of surface tension. The solidification of the valley results in the occurrence of a second swelling. This process repeats and humping defect forms. Marangoni force and viscous force also have influence on this process. In addition, it is found that adding a Tungsten Inert Gas arc behind the laser beam can effectively suppress the humping.

## 1. Introduction

Because of the high-power density of a laser beam, generally laser welding has higher welding speed than arc welding, and thus can achieve higher productivity [1,2,3,4]. However, the productivity of laser welding cannot be improved without limit by simply increasing the welding speed and laser power proportionately. It is found that with the increase of welding speed and laser power, humping defects, which can be described as periodic undulations consisting of a series of swellings and valleys, occur easily on the weld seam [5,6,7]. The occurrence of humping severely deteriorates the product properties, and limits the further increase of welding speed.

Studies have been performed to understand the formation mechanism of humping in high-speed laser welding. Some researchers investigated the behavior of the melt pool in high-speed laser welding through high-speed imaging experiments and numerical simulations and explained the formation of humping with the Rayleigh instability model [8,9,10,11,12,13]. The Rayleigh instability model is a common explanation for the formation of humping. According to this theory, the melt flow in the melt pool is analogous to a cylindrical inviscid fluid jet freely suspended in space, and the lateral surface of the cylindrical jet becomes unstable once its length *L* exceeds its circumference 2π*R*. With the existence of tiny perturbations, the radius of the jet becomes non-uniform, so the additional pressure caused by surface tension also becomes non-uniform, leading to the periodic swelling and narrowing of the jet. This theory can explain the formation of humping to some extent, such as the periodic occurrence of swelling and narrowing in the melt pool, but it is a highly idealized model, and many important factors influencing the dynamic behavior of melt pool are ignored, such as the effects of viscosity and solidification. Hence, there is an ongoing debate about the validity of this explanation, and it needs further improvement [14].

There are also some other explanations for humping formation in high-speed laser welding. Kawahito et al. [15] observed the melt pool behavior through high-speed imaging and believed that the humping formation was dependent upon several dynamic or static factors such as melt volume, strong melt flow, solidification, and surface tension. Ai et al. [16] speculated that the collision of the backflow with the fast rearward melt flow provided the condition for the wave formation in the melt pool, which led to the occurrence of humping. Though these explanations were proposed according to the observation of the melt pool image, they were still vague. The humping formation process was still not described in detail. Therefore, the formation mechanism of humping in high-speed laser welding still needs further research.

In order to improve the productivity of laser welding while maintaining product quality, some methods have been proposed to suppress humping, but existing methods are still limited. Kawahito et al. [15] found that humping could be reduced by performing inner defocused welding, but the penetration depth would decrease in this way. Cornell et al. [17] used laser wobble welding to reduce humping, but this method only mitigated the undulation of the weld seam surface, and humping was not suppressed completely. 

In this research, high-speed imaging experiment and numerical simulation of high-speed laser welding process are performed. Through the analysis of the experimental and simulation results, the humping formation process is explained. In addition, the effects of surface tension, Marangoni force, and viscous force on humping formation are studied. Finally, based on the analysis of the humping formation mechanism, a humping suppression method is proposed by adding a TIG (Tungsten Inert Gas) arc behind the laser beam, and humping is suppressed successfully in this way.

## 2. Experimental Setup and Procedure

**Figure 1:**
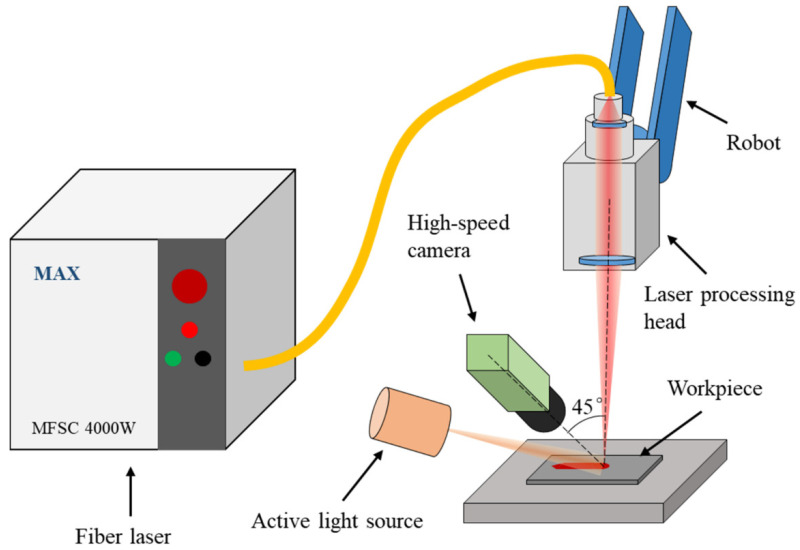
Experiment configuration.

A high-speed imaging experiment is conducted to observe the dynamic behavior of the melt pool in high-speed laser welding. The welding method is bead-on-plate welding. The workpieces are 304 stainless steel sheets with the following dimensions: length 200 mm × width 50 mm × thickness 1 mm. Before the welding process, the surfaces of workpieces are polished with sandpaper and cleaned with absolute ethyl alcohol. A MFSC-4000W single-mode fiber laser (MAX, Shenzhen, China) and a YC52 laser processing head (PRECITEC, Gaggenau, Germany) are integrated to output a laser beam. The maximum power of the laser is 4000 W, and the wavelength of the output laser is 1070 ± 10 nm. The diameter of the laser spot is 0.4 mm. The laser processing head is installed on a MH24 robot (YASKAWA, Kitakyushu, Japan). Argon is used as the shielding gas, and its flowrate is 20 L/min. A Memrecam HX-6 high-speed camera (NAC, Tokyo, Japan)is used to capture images of the melt pool during the welding process, and its frame rate is set to 5 kHz. The axis of the camera forms a 45° angle with the horizontal plane. Since the reflected laser and bright laser-induced vapor plume in the image can disturb the observation of the melt pool, a CAVILUX HF pulsed high power diode laser light source (CAVITAR, Tampere, Finland) is used as an active light source for illumination, and its central wavelength is 810 nm, and a narrow band-pass filter with the central wavelength of 810 nm is attached on the camera lens. In this way, the interference factors in the image can be weakened, and the melt pool can be clearer. The configuration of the experiment is shown in Figure 1.

Welding parameters used in the experiment are listed in Table 1. The linear energy density of the laser is kept consistent for the convenience of comparing the behavior of the melt pool with similar penetration depth. 

## 3. Numerical Simulation Model

### 3.1. Assumptions and Governing Equations

In order to understand the dynamic behavior of the melt pool in high-speed laser welding in detail, a simulation model is built. In the simulation, fluids are assumed to form an incompressible laminar flow with Newtonian viscosity. Solutions are found for the governing equations of mass conservation, momentum conservation (Navier–Stokes), and energy conservation in the simulation.

Mass conservation equation:

(1) $$\frac{\partial \rho }{\partial t}+\nabla \cdot \left(\rho \vec{v}\right)=0$$

Momentum conservation equation:

(2) $$\frac{\partial }{\partial t}\left(\rho \vec{v}\right)+\nabla \cdot \left(\rho \vec{v}\vec{v}\right)=-\nabla p+\nabla \cdot \left(\mu \nabla \vec{v}\right)+\vec{S_{f}}$$

Energy conservation equation:

(3) $$\frac{\partial }{\partial t}\left(\rho H\right)+\nabla \cdot \left(\vec{v}\rho H\right)=\nabla \cdot \left(k\nabla T\right)+S_{h}$$

where ρ, $\vec{v}$, p, μ, $\vec{S_{f}}$, H, k, T, and $S_{h}$ are density, velocity, pressure, dynamic viscosity, external body force, enthalpy, thermal conductivity, temperature, and heat sources of fluids, respectively.

In addition, the multiphase flow problem is involved since both the gas phase and liquid phase of fluids are included in the simulation, so the VOF (Volume of Fluid) method is adopted to model the multiphase flow problem. VOF equation:

(4) $$\frac{\partial }{\partial t}\left(\alpha_{i}\rho_{i}\right)+\nabla \cdot \left(\alpha_{i}\rho_{i}{\vec{v}}_{i}\right)=\dot{m_{i}}$$

where the subscript i represents the i-th phase of fluids, $\alpha_{i}$ is the volume of fluid of the i-th phase, the sum of $\alpha_{i}$ for all phases is 1, and $\dot{m_{i}}$ is the mass change of the i-th phase caused by phase transformation. 

### 3.2. Melting and Solidification 

The enthalpy-porosity technique [18] is adopted to model melting and solidification. The solid–liquid interface is not traced explicitly. A variable called liquid fraction β is introduced, and it represents the ratio of the liquid phase in the cell. The enthalpy of the material is composed of sensible enthalpy h and latent heat ∆H:

(5) H=h+∆H

where

(6) $$h=h_{\mathrm{ref}}+\int_{T_{\mathrm{ref}}}^{T}c_{p}dT$$

(7) ∆H=βL

where $h_{\mathrm{ref}}$ is the reference enthalpy, $c_{p}$ is the specific heat, L is the latent heat of the material, and β is defined as

(8) $$\beta =\{\begin{array}{c} 0,\;\;\;\;\;\;\;\mathrm{if}\;T<T_{s}\\ 1,\;\;\;\;\;\;\;\mathrm{if}\;T>T_{l}\\ \frac{T-T_{s}}{T_{l}-T_{s}},\;\mathrm{if}\;T_{s}<T<T_{l} \end{array}$$

where $T_{s}$ and $T_{l}$ are the solidus temperature and the liquidus temperature of the material, respectively.

The momentum source due to the solidification and melting is given as

(9) $$\vec{S_{f-\mathrm{ms}}}=\frac{{\left(1-\beta \right)}^{2}}{\left(\beta^{3}+C_{K}\right)}C_{\mathrm{mush}}\vec{v}$$

where $C_{K}$ is a small number to prevent division by zero, and $C_{\mathrm{mush}}$ is the mushy zone constant.

### 3.3. Vaporization and Condensation

The condensation process is thought to have little influence on the fluid flow, so it is ignored in the simulation. The mass loss of the liquid phase due to vaporization is written as [19]

(10) $$q_{v}=0.82\frac{L_{v}M}{\sqrt{2\pi MRT}}P_{0}\exp\left[\frac{L_{v}M\left(T-T_{v}\right)}{RTT_{v}}\right]$$

where $L_{v}$ is the latent heat of vaporization, $T_{v}$ is the boiling temperature, M is the molar mass, R is the universal gas constant, and $P_{0}$ is the ambient pressure.

### 3.4. Surface Tension and Recoil Pressure

The pressure difference between the liquid and gas on either side of the free surface due to surface tension is

(11) $$P_{\sigma }=\sigma K$$

where K is the surface curvature, and σ is the surface tension coefficient:

The surface tension coefficient is calculated as [12,20,21]

(12) $$\sigma =\sigma_{0}+A\left(T-T_{s}\right)-RT\left[\Gamma_{S}\ln\left(1+K_{S}\alpha_{S}\right)+\Gamma_{O}\ln\left(1+K_{O}\alpha_{O}\right)\right]$$

where $\sigma_{0}=1.77\;N\cdot m^{-1}$ is the surface tension coefficient of pure SUS304 stainless steel at the solidus temperature, $A=-0.43\times 10^{-3}\;N\cdot m^{-1}\cdot K^{-1}$ is the temperature coefficient of surface tension coefficient of pure SUS304 stainless steel, $\Gamma_{S}=1.3\times 10^{-5}\;\mathrm{mol}\cdot m^{-2}$ and $\Gamma_{O}=2.03\times 10^{-5}\;\mathrm{mol}\cdot m^{-2}$ are the surface excesses at saturation of sulfur and oxygen, respectively, $K_{S}=k_{1,S}e^{\left(\frac{-∆H_{S}}{RT}\right)}$ and $K_{O}=k_{1,O}e^{\left(\frac{-∆H_{O}}{RT}\right)}$ are the equilibrium constants for segregation of sulfur and oxygen, respectively, $k_{1,S}=0.00318$ and $k_{1,O}=0.0138$ are the entropy factors of sulfur and oxygen, respectively, $∆H_{S}=-1.66\times 10^{5}\;J\cdot {\mathrm{mol}}^{-1}$ and $∆H_{O}=-1.463\times 10^{5}\;J\cdot {\mathrm{mol}}^{-1}$ are the standard heat of absorption of sulfur and oxygen, respectively, $\alpha_{S}=0.03$ and $\alpha_{O}=0.01$ are the weight percentages of soluble sulfur and oxygen, respectively. According to Equation (12), the temperature coefficient of surface tension coefficient is

(13) $$\begin{array}{cc} \frac{d\sigma }{dT} & =A-R\Gamma_{S}[\ln\left(1+K_{S}\alpha_{S}\right)+\frac{K_{S}\alpha_{S}}{1+K_{S}\alpha_{S}}\frac{∆H_{S}}{RT}]\\ & \;-R\Gamma_{O}[\ln\left(1+K_{O}\alpha_{O}\right)+\frac{K_{O}\alpha_{O}}{1+K_{O}\alpha_{O}}\frac{∆H_{O}}{RT}] \end{array}$$

**Figure 2:**
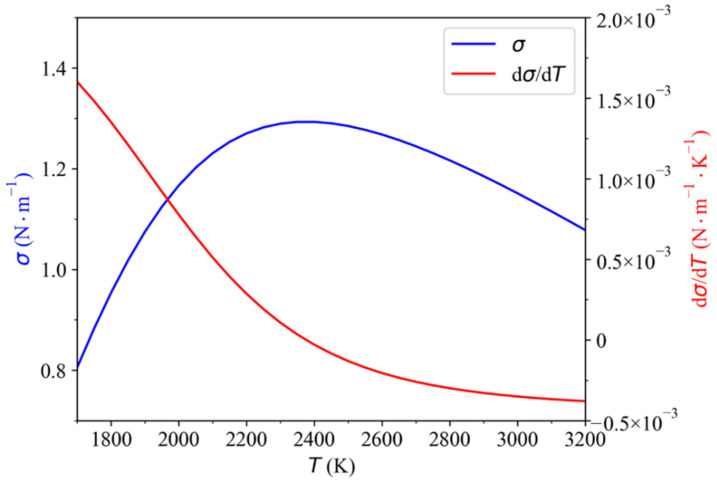
The dependence of σ and dσ/dT on temperature.

The dependence of σ and dσ/dT on temperature is shown in Figure 2. It can be found that the value of dσ/dT is positive when the temperature is below about 2300 K, and it turns negative with the further increase of temperature.

The recoil pressure is calculated as follows

(14) $$P_{r}=0.54P_{0}exp\left[\frac{L_{v}M\left(T-T_{v}\right)}{RTT_{v}}\right]\;$$

### 3.5. Boundary Condition

The heat boundary condition of the top free surface is influenced by laser heat flux, the heat dissipation by convection and radiation, and the heat loss by vaporization:

(15) $$K\frac{\partial T}{\partial \vec{n}}=q_{\mathrm{laser}}-h_{A}\left(T-T_{\infty }\right)-\varepsilon_{r}\sigma_{s}\left(T^{4}-T_{\infty }^{4}\right)-q_{v}$$

where $q_{\mathrm{laser}}$ is the absorbed laser energy by the workpiece, $h_{A}$ is the convection coefficient, $T_{\infty }$ is the ambient temperature, $\varepsilon_{r}$ is the emissivity, $\sigma_{s}$ is the Stefan–Boltzmann constant, and $q_{\mathrm{vap}}$ is the heat loss due to vaporization.

For the heat boundary conditions of other free surfaces, only the heat dissipation by convection and radiation are taken into consideration.

### 3.6. Laser Heat Source

The surface power distribution of the laser is assumed to be a Gaussian function which has a mathematical form like the following equation

(16) $$q\left(x,y,t\right)=\frac{3Q}{\pi r_{0}^{2}}\exp\left[-3\frac{{\left(x-x_{0}-u_{w}t\right)}^{2}+y^{2}}{r_{0}^{2}}\right]$$

where Q is the total laser power, the coefficient 3 means that 95% of the laser power exists in the area of radius $r_{0}$, $r_{0}=0.2\;\mathrm{mm}$ denotes the focal radius of the laser beam, $x_{0}$ is the welding starting position on the *x* axis, and $u_{w}$ is the welding speed along *x* axis. In each step of the calculation, the free surface between metal and gas is searched, and then the laser heat is applied on corresponding cells.

### 3.7. Numerical Consideration

**Figure 3:**
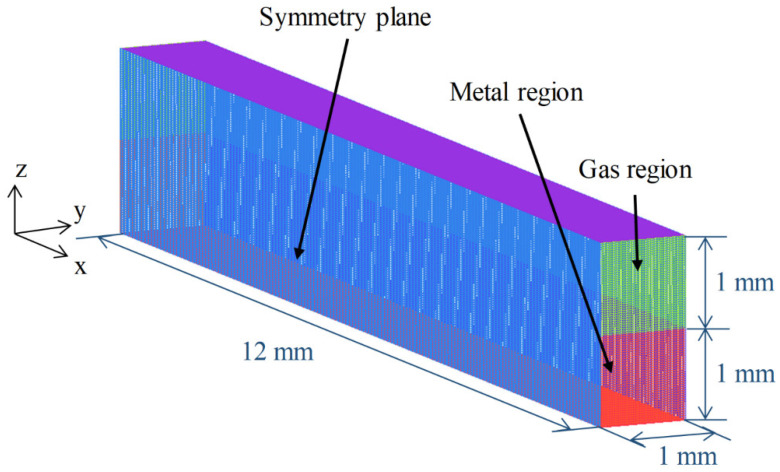
Calculation domain.

A symmetry calculation domain is built and its size is length 12 mm × width 1 mm × height 2 mm. The heights of the metal zone and the gas zone are both 1 mm, as shown in Figure 3. The calculation domain is divided into uniform cubic grids with edge length of 0.025 mm, so the total number of grids is 1,536,000. The welding direction is along x axis and the starting and ending positions are *x* = 1.5 mm and *x* = 11.5 mm, respectively. The physical property parameters of SUS304 stainless steel and other parameters used in the simulation are listed in Table 2. The commercial computational fluid dynamics software ANSYS Fluent is adopted, and the PISO (Pressure-Implicit with Splitting of Operators) scheme is chosen to solve this model. The time step is set to 1 × 10^−6^ s. The simulation program runs on the High Performance Computing (HPC) cluster supported by the Center of High performance computing, Tsinghua University [22]. 56 Intel(R) Xeon(R) CPU E5-2680 v4 processors are used in parallel. 

## 4. Results and Discussion

### 4.1. Validation of the Simulation Model

**Figure 4:**
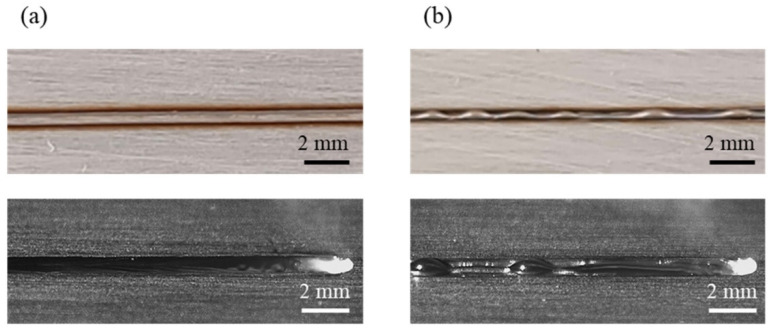
Appearances of the weld seam (**top**) and melt pool (**bottom**) under different welding parameters in the experiments: (**a**) 2000 W, 16 m/min, (**b**) 3000 W, 24 m/min.

**Figure 5:**
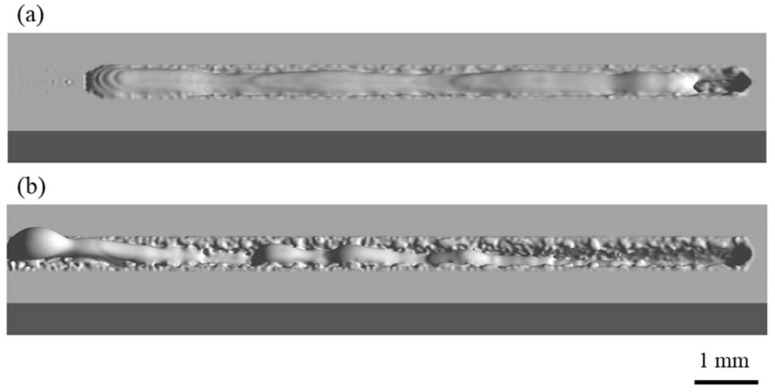
Appearances of the weld seam under different welding parameters in the simulations: (**a**) 2000 W, 16 m/min, (**b**) 3000 W, 24 m/min.

**Figure 6:**
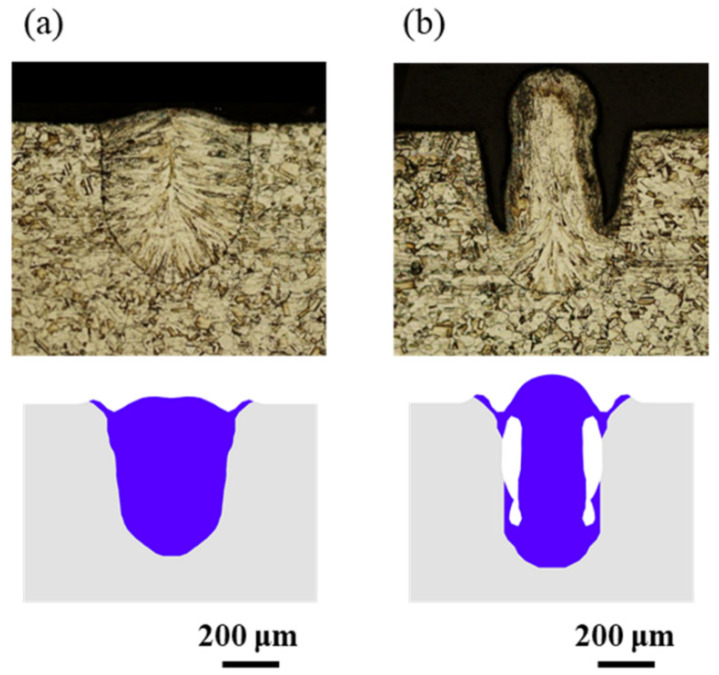
Comparison of the weld seam cross section between experiments (**top**) and simulations (**bottom**): (**a**) 2000 W, 16 m/min, (**b**) 3000 W, 24 m/min.

Appearances of the weld seam and melt pool at different welding parameters in the experiments are shown in Figure 4. It can be found that at a welding speed of 16 m/min, no humping occurs on the weld seam, and the melt pool is relatively short and flat. At a welding speed of 24 m/min, humping occurs on the weld seam, and the melt pool is long, and obvious swellings appear in it. Two simulation cases are performed using welding parameters in Table 1, and appearances of the weld seam in the simulation results are shown in Figure 5. It can be found that no humping occurs at a welding speed of 16 m/min, and humping occurs at a welding speed of 24 m/min, which agrees with the experimental results. In addition, cross sections of the weld seams in experiments and simulations are given in Figure 6, and width and depth of the weld seams are listed in Table 3, and the simulation results are close to the experiment results. Therefore, the validity of the simulation model is verified.

### 4.2. Melt Pool Behavior and Humping Formation

**Figure 7:**
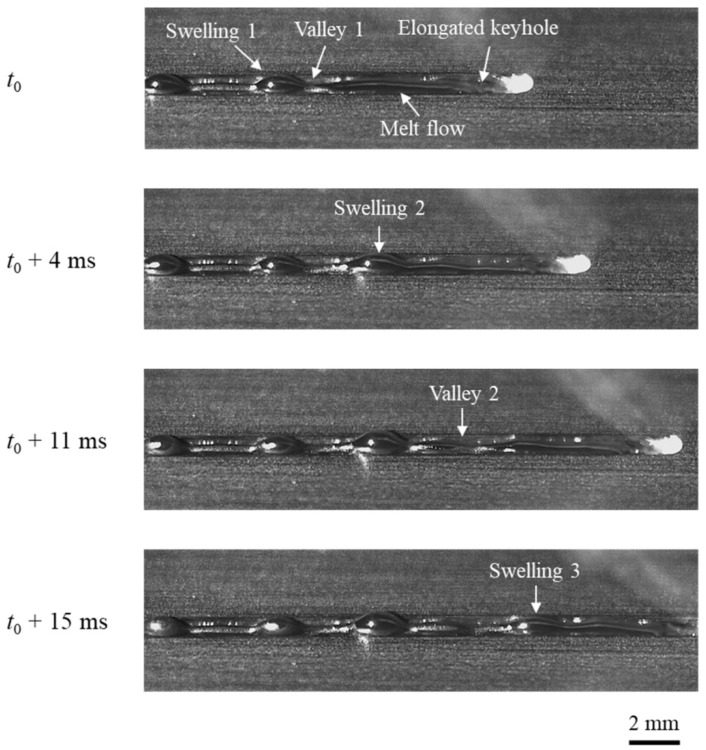
Humping formation process in the experiment with welding parameters of 3000 W, 24 m/min.

The behavior of melt pool during humping formation process at welding speed of 24 m/min is observed through high-speed imaging, as shown in Figure 7. In the image at *t*_0_, it can be observed that a long and narrow groove exists behind the laser irradiation region. Fabbro [10] also noticed this phenomenon and referred to this groove as “elongated keyhole”. In addition, the melt pool is also elongated and high-speed melt flow can be noticed. A swelling of molten metal (swelling 1) exists at the tail of the melt pool. The melt flow in front of this swelling is thin and forms a valley (valley 1). Most of the melt here has solidified, and the flow channel has narrowed obviously. At *t*_0_ + 4 ms, molten metal accumulates in front of valley 1 and forms a new swelling (swelling 2). At *t*_0_ + 11 ms, a new valley (valley 2) appears in front of swelling 2, and the melt here solidifies earlier than swelling 2. At *t*_0_ + 15 ms, molten metal accumulates in front of valley 2 and forms a new swelling (swelling 3). Swellings and valleys appear alternately, and then, humping forms after the melt pool solidifies.

**Figure 8:**
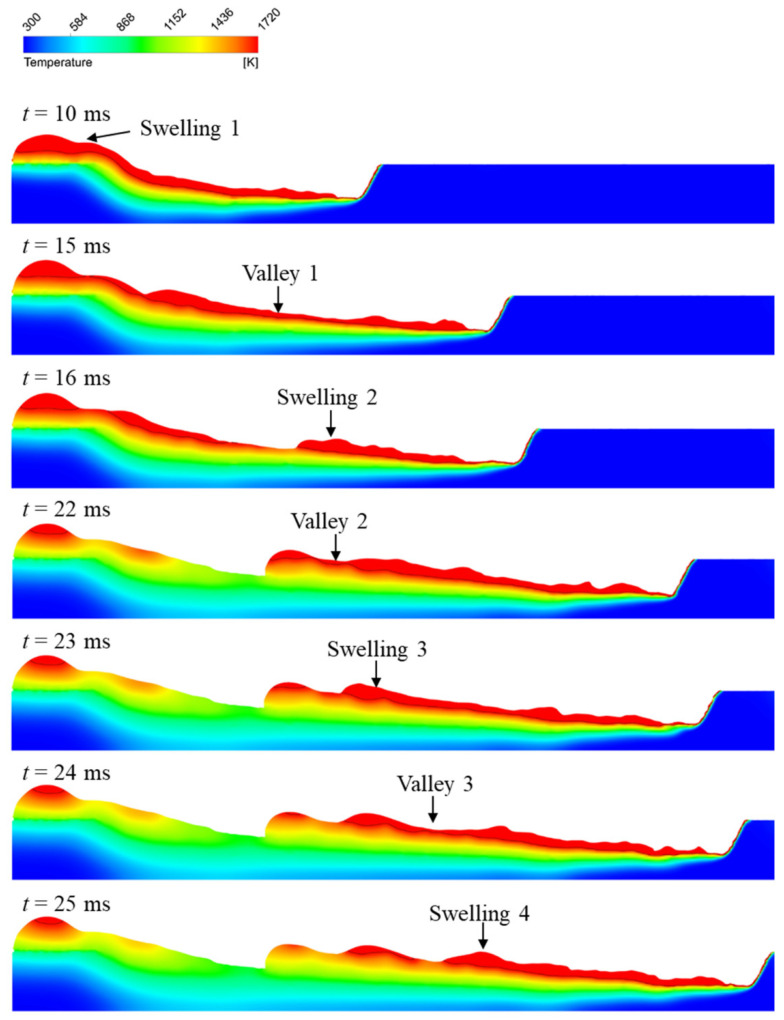
Humping formation process in the simulation result with welding parameters of 3000 W, 24 m/min.

The humping formation process in the simulation result is similar to that in the experimental result. The evolution of the longitudinal section of the melt pool during the humping formation process is shown in Figure 8, and the black curves denote the isothermal contours of solidus temperature. After the welding process starts, high-speed melt flow appears in the melt pool, and the keyhole and melt pool are long. At 10 ms, the molten metal flowing out of the melt pool accumulates on the surface of the workpiece and forms a swelling (swelling 1). At 15 ms, a thin layer (valley 1) appears in the melt pool. At 16 ms, the molten metal at valley 1 solidifies, and a new swelling (swelling 2) starts to form in front of valley 1. At 22 ms, swelling 2 has grown up and a new valley (valley 2) appears in front of swelling 2. Then, swelling 3, valley 3 and swelling 4 appear successively.

From the above observation, it can be found that there are two important phenomena in the humping formation process: the high-speed melt flow and the alternate appearance of swellings and valleys. Next the flow of the melt pool in the simulation result is analyzed to explain the cause of these two phenomena. 

**Figure 9:**
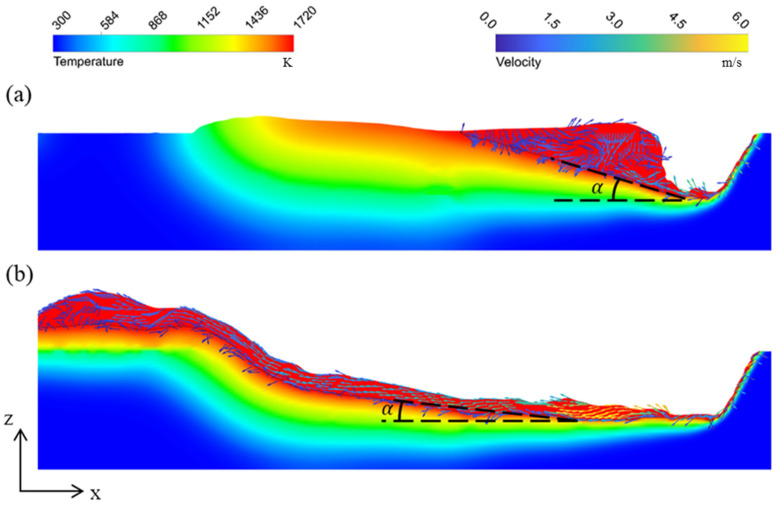
Velocity fields on the longitudinal section of the melt pool in the simulation results: (**a**) 2000 W, 16 m/min; (**b**) 3000 W, 24 m/min.

**Figure 10:**
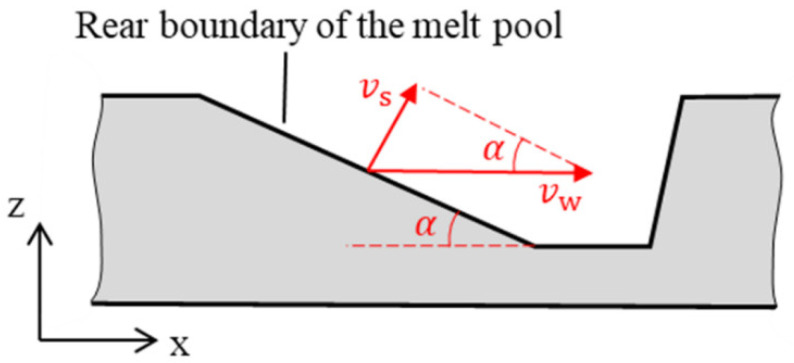
Model of the melt pool rear boundary.

First the cause of the high-speed melt flow is analyzed. The velocity fields on the longitudinal section of the melt pool at different welding speeds are compared in Figure 9. It can be observed that at welding speed of 24 m/min the melt flow has higher velocity, and the maximum velocity can reach 6 m/s or higher. In addition, shapes of the melt pool are much different between these two simulation cases. At a welding speed of 16 m/min, the melt pool is short and thick, but with a welding speed of 24 m/min, the melt pool is long and thin. The differences of melt flow velocity and melt pool shape can be attributed to two reasons. The first reason is the different recoil pressure. The recoil pressure is the main driving force of the melt flow and is influenced by the temperature of the keyhole front wall as Equation (14) shows. The maximum temperatures of the keyhole front wall in these two simulation cases are 3547 K and 3805 K for welding speeds of 16 m/min and 24 m/min, respectively. With the same heat input, the keyhole front wall has less cooling time under higher welding speed, and thus, it has higher temperature. Therefore, it has larger recoil pressure, and therefore, the melt flow has a higher initial velocity when leaving the keyhole front wall. The other reason is the tilt angle *α* of the melt pool rear boundary. As shown in Figure 10, *α* can be calculated as

(17) $$\sin\alpha =\frac{v_{s}}{v_{w}}$$

where *v*_s_ is the moving speed of the melt pool rear boundary, and *v*_w_ is the welding speed. When the welding speed is high, *α* is small. That is to say that the melt pool rear boundary has a gentle slope, which can also be observed in Figure 9. When the melt flow reaches the melt pool rear boundary, a smaller *α* can help it to retain a high *x*-direction velocity component and flow rearward, and thus, the high-speed melt flow can be maintained.

**Figure 11:**
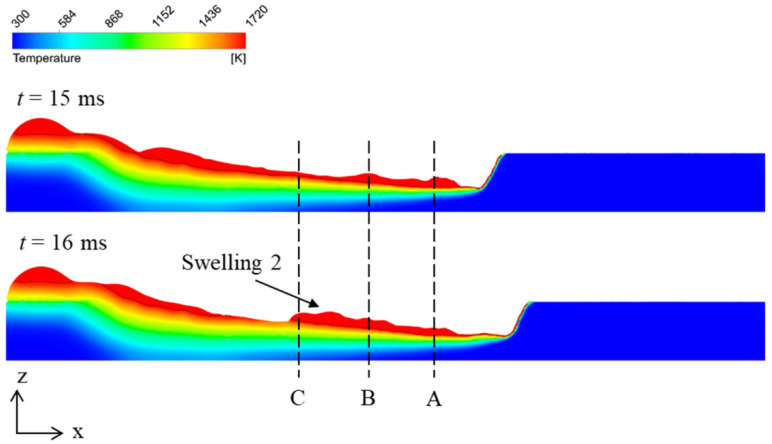
Illustration of measuring cross sections (taking swelling 2, for example).

Next, the cause of the alternate appearance of swellings and valleys is analyzed. In Figure 8, at the beginning of the welding process, the molten metal flows out from the melt pool to the surface of the workpiece and then cools down and solidifies rapidly. During the solidification, the viscosity of the molten metal increases sharply [23], so the molten metal accumulates and swelling 1 forms. At this time, the melt pool has just formed, so the formation process of swelling 1 lacks generality. Therefore the formation of the subsequent swellings and valleys is the main focus below. In order to analyze the flow of the melt pool quantitatively, for every swelling except swelling 1 in Figure 8, three measuring cross sections are picked in the melt pool at the moment 1 ms before the swelling starts to form, and the flowrates along the minus x direction on these cross sections are measured. For these three cross sections, one is 1.5 mm behind the laser beam center and denoted as cross section A, one is at the tail of the swelling when it starts to form and denoted as cross section C, and one is 1 mm in front of cross section C and denoted as cross section B. For example, as shown in Figure 11, for swelling 2, *t* = 16 ms is regarded as the moment it starts to form, so the flowrates on cross sections A, B, and C at *t* = 15 ms are measured. Then, the changes of flowrate per unit length ∆q/∆x between two adjacent cross sections are calculated. The positions of measuring cross sections and the measurement results of flowrates for different swellings are listed in Table 4. It can be noticed that at the moment 1 ms before swellings start to form, the absolute value of ∆q/∆x between B and C is much larger than that between A and B. The average value of ∆q/∆x is −15.6 mm^2^/s between B and C, while −5.8 mm^2^/s between A and B. This indicates that the melt flow decelerates more sharply between B and C, leading to the accumulation of molten metal, so the swelling starts to form. The sharp deceleration of the melt flow is caused by the solidification of the melt pool. As shown in Figure 11, before the swelling starts to form, the melt flow at C is thin, so the heat capacity is small, and the melt pool solidifies rapidly here. The subsequent melt flow is hindered by the sharply increasing viscosity and decelerates when reaching the solidified region near C, so molten metal accumulates, and the swelling starts to form here. This process also agrees with the observed process in the experiment, in which a valley appears and solidifies rapidly in the melt pool and then a swelling appears in front of the valley. 

When a swelling starts to form, the radius of the melt pool at the swelling increases. Because the melt pool is thin and long, it is unstable under the effect of surface tension, as described in the Rayleigh instability model. The additional pressure due to surface tension decreases at the swelling. Therefore, the molten metal in front of the swelling flows into the swelling further, resulting in the further growing up of the swelling. Meanwhile, the melt flow in front of the swelling narrows, leading to the formation of a valley. Then, the valley solidifies and induces the formation of the next swelling.

It should be pointed out that the proposed explanation here has similarity to the traditional explanation of humping formation by using Rayleigh instability model [8,9,10,11,12,13]. The difference is that, in the traditional explanation, the origin of the non-uniform radius of the melt pool is attributed to perturbations, which is vague. In the proposed explanation here, the origin of the non-uniform radius of the melt pool is made clear. The solidified region in the melt pool hinders the subsequent melt flow, leading to the accumulation of molten metal, so the radius of the melt pool becomes non-uniform. 

### 4.3. Effect of Surface Tension and Marangoni Force

**Figure 12:**
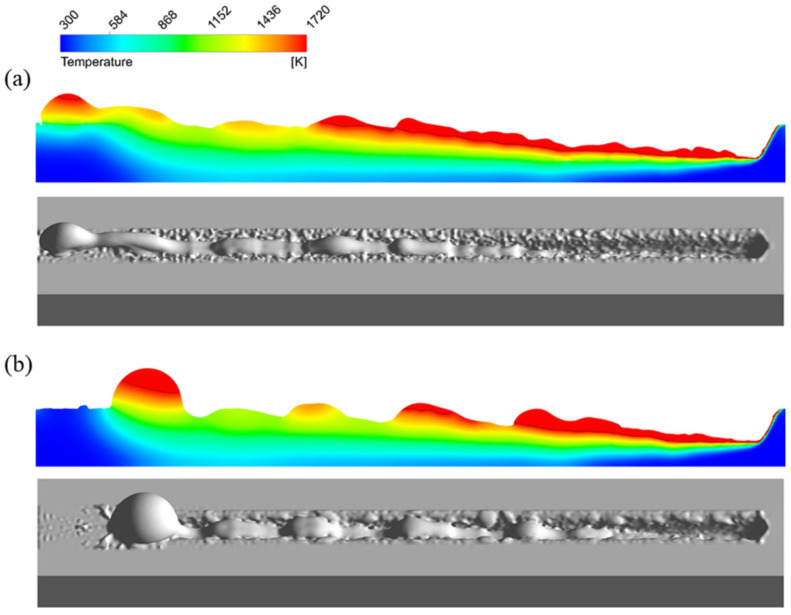
Longitudinal sections (**top**) and appearances (**bottom**) of the simulated weld seams (3000 W, 24 m/min) while dσ/dT remains unchanged: (**a**) $\sigma_{0}=1.27\;N\cdot m^{-1}$, (**b**) $\sigma_{0}=2.27\;N\cdot m^{-1}$.

**Figure 13:**
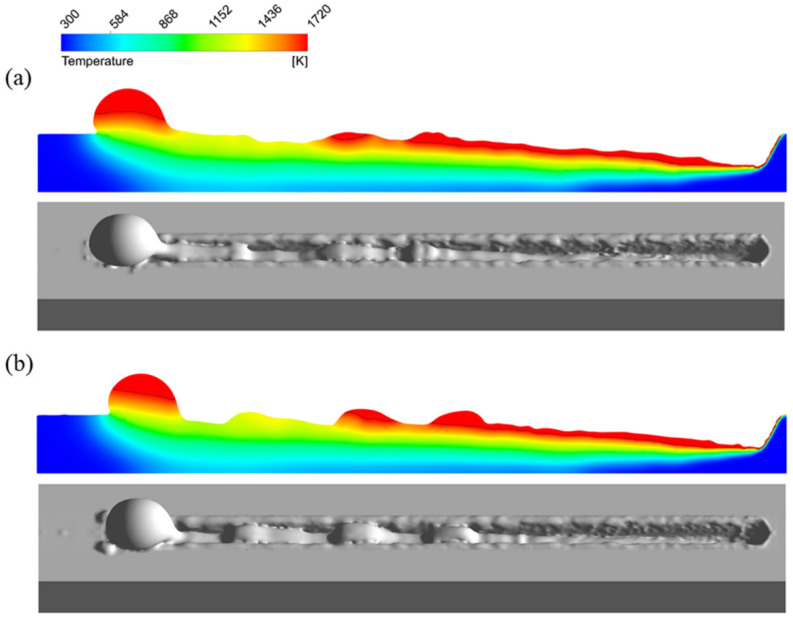
Longitudinal sections (**top**) and appearances (**bottom**) of the simulated weld seams (3000 W, 24 m/min) while dσ/dT=0: (**a**) $\sigma =1.26\;N\cdot m^{-1}$, (**b**) $\sigma =1.76\;N\cdot m^{-1}$.

From the above analysis, it can be noticed that the surface tension plays an important role in the humping formation process, so it is necessary to investigate the effect of surface tension. In addition, the temperature gradient of surface tension produces a Marangoni force, so the effect of the Marangoni force also needs investigation. The welding parameters used here are still 3000 W and 24 m/min. In the simulation model, the surface tension coefficient σ is determined by $\sigma_{0}$ and dσ/dT, as shown in Equation (12). First $\sigma_{0}$ is changed from its original value $1.77\;N\cdot m^{-1}$ to $1.27\;N\cdot m^{-1}$ and $2.27\;N\cdot m^{-1}$, respectively, while dσ/dT remains unchanged. The temperatures of most regions in the melt pool are below 2000 K except the region near the laser beam, so dσ/dT can be regarded as positive. The longitudinal sections and appearances of the simulated weld seams are shown in Figure 12. Next dσ/dT is fixed to 0. That is to say, σ is set to fixed values. σ is set to $1.26\;N\cdot m^{-1}$ and $1.76\;N\cdot m^{-1}$, respectively, and the longitudinal sections and appearances of the simulated weld seams are shown in Figure 13.

From Figure 12 and Figure 13, it can be found that with the increase of σ, the humping becomes more severe. This is due to the fact that when σ increases, the difference of additional pressure between the swelling and the melt flow in front of it increases, so it is easier for the swelling to grow and for a valley in front of the swelling to form and induce the formation of the next swelling. 

**Figure 14:**
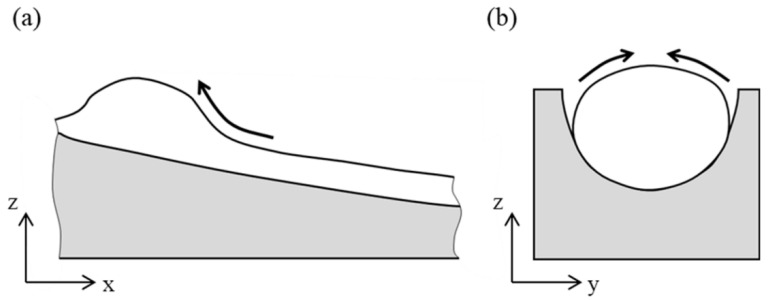
Direction of the Marangoni force in the melt pool: (**a**) on the longitudinal section, (**b**) on the cross section.

In addition, in the simulation case of Figure 5b, when *T <* 2000 K, it can be calculated that $\sigma <1.16\;N\cdot m^{-1}$. That is to say that σ in Figure 5b is smaller than that in Figure 13a where $\sigma =1.16\;N\cdot m^{-1}$. According to the above analysis, a smaller σ can help to reduce humping. However, it can be found that humping in Figure 5b is more severe than that in Figure 13a. This indicates that a positive dσ/dT can contribute to the formation of humping. When dσ/dT>0, the Marangoni forces pointing from low temperature region to high temperature region exist in the melt pool. The center of the swelling has a large heat capacity, so the temperature here is higher than in the surrounding region. On the longitudinal section of the melt pool, the Marangoni force points from the front to the swelling, as shown in Figure 14a. On the cross section of the swelling, the Marangoni force points from sides to the center, as shown in Figure 14b. The Marangoni forces on these two sections can both contribute to the growing up of the swelling and thus make humping more severe.

### 4.4. Effect of Viscous Force

**Figure 15:**
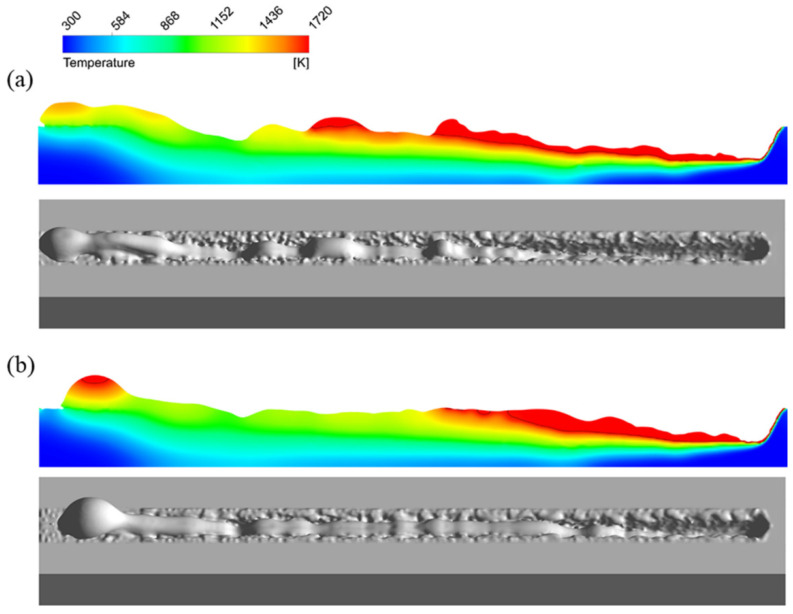
Longitudinal sections (**top**) and appearances (**bottom**) of the simulated weld seams (3000 W, 24 m/min) with different viscosity: (**a**) μ=0, (**b**) μ=0.02.

The viscous force can influence the deceleration of the melt flow, so it shall also influence the formation of humping. In order to investigate the effect of the viscous force, the viscosity of the liquid metal μ is changed from its original value of 0.006 to 0 and 0.02, respectively. The longitudinal sections and appearances of the simulated weld seams are shown in Figure 15. With the increase of the viscosity, the appearance of the weld seam becomes flat, and humping becomes less severe. According to the previous analysis of the humping formation process, the high-speed melt flow is hindered by the solidified region in the melt pool, leading to the accumulation of molten metal and the formation of the swelling, which is the origin of the humping formation process. With large viscosity, the melt flow has decelerated significantly under the effect of viscous force before reaching the solidified region in the melt pool, making it harder for the swelling to form, so humping becomes less severe. This result can also be a reference to humping suppression. If some methods can be taken to influence the behavior of the melt pool and make the high-speed melt flow decelerate in advance, then humping can be suppressed.

## 5. Humping Suppression by TIG Arc

**Figure 16:**
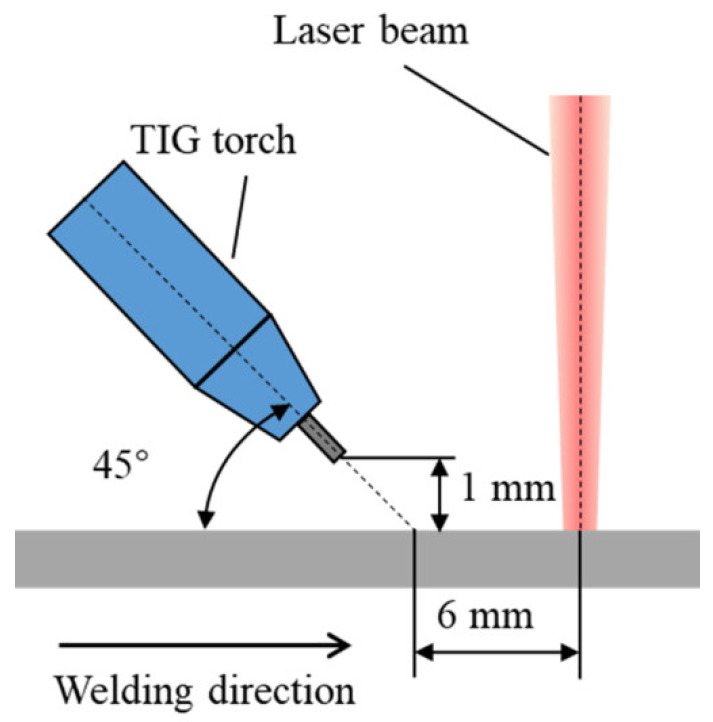
Position of the TIG torch.

According to the above analysis, a humping suppression method is proposed by adding a TIG (Tungsten Inert Gas) arc behind the laser beam. An experiment is conducted to investigate the influence of adding a TIG arc behind the laser beam on humping formation in high-speed laser welding. The experimental setup described in Section 2 is used to perform laser welding and high-speed imaging. The laser power is 3000 W, and the welding speed is 24 m/min during the experiment. In addition, a Fronius TransTig 4000 TIG power source is used to output the TIG arc, and the direct current electrode negative (DCEN) setup is used. The arc current is 100 A. The TIG torch is fixed on the laser processing head, and the relative position relationship between the torch and the laser beam is shown in Figure 16. The flowrate of the shielding gas Argon delivered out of the TIG nozzle is 8 L/min. The active light source is not used during this experiment since its light can be blocked by the TIG torch. 

**Figure 17:**
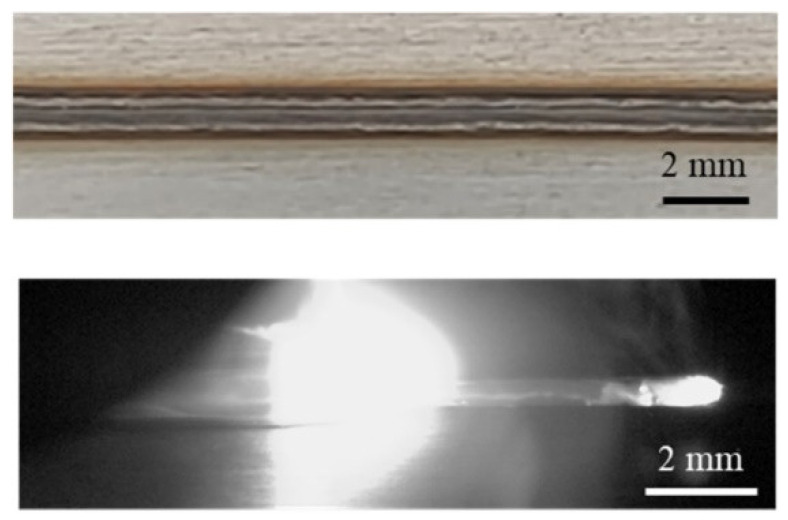
Appearances of the weld seam and melt pool (3000 W, 24 m/min) with TIG arc added.

**Figure 18:**
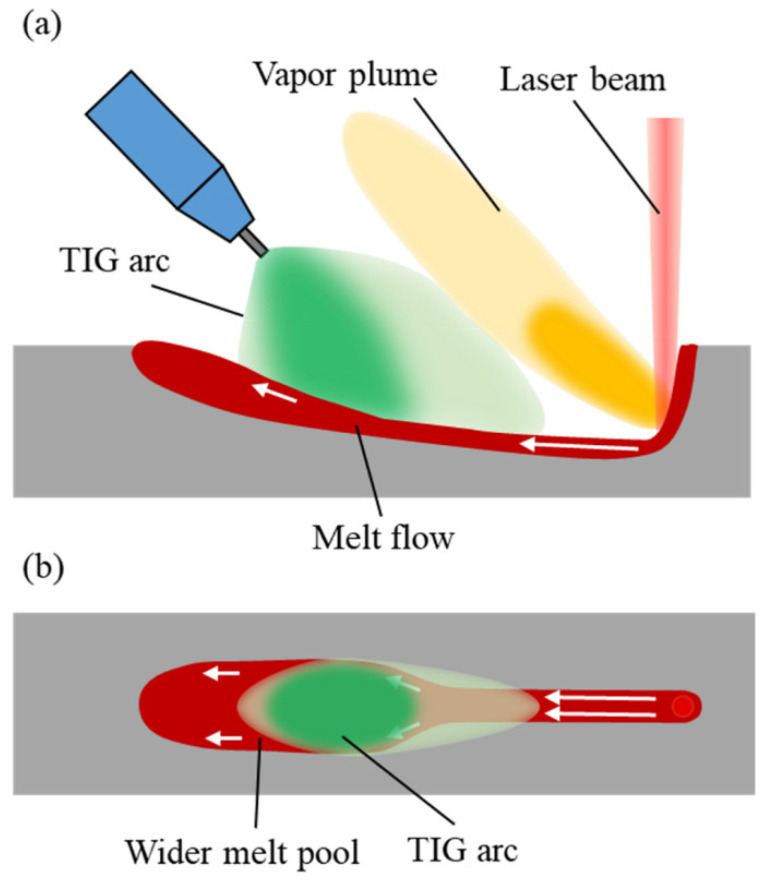
Melt pool behavior assisted by TIG arc: (**a**) on the longitudinal section, (**b**) on the melt pool surface.

Appearances of the weld seam and melt pool with TIG arc added are shown in Figure 17. No humping occurs on the weld seam, indicating that humping in high-speed laser welding can be suppressed by adding a TIG arc behind the laser beam. The arc and laser beam act on a common melt pool, and the melt pool becomes wider after going through the arc, and the surface of the melt pool is flat. Due to this, the arc force and arc heat can influence the behavior of the melt pool. Because the distance between the laser beam and the TIG electrode is relatively large, the coupling of the arc with the laser-induced vapor plume does not occur. The laser irradiation region does not get heat from the arc, so the melt flow is not strengthened by the arc, which is different from the typical laser-arc hybrid welding [24,25]. Due to the inclination angle of the TIG torch, the arc force becomes the resistance of the high-speed melt flow, and therefore, the melt flow decelerates significantly before reaching the solidified region in the melt pool, as shown in Figure 18a. In addition, under the effect of the arc heat, the melt pool becomes wider after going through the arc, as shown in Figure 18b. The increase of the melt pool width can make the melt flow decelerate further in case of flow conservation and can also reduce the instability of the melt flow. Therefore, when the melt flow reaches the solidified region of the melt pool, its sharp deceleration is avoided, so the swelling does not occur. As a result, humping can be suppressed. 

## 6. Conclusions

In this research, a detailed study was conducted to investigate the humping formation and suppression in high-speed laser welding through high-speed imaging and numerical modeling. The main conclusions are as follows:

(1) At high welding speed, the high-speed rearward melt flow caused by recoil pressure is hindered by the solidified region and accumulates, causing the formation of swelling. Then, under the effect of surface tension, the swelling grows. This process repeats leading to humping.
(2) The increase in surface tension and Marangoni force can lead more molten metal to flow into the swelling and promote the humping formation process.
(3) The increase in viscosity of the molten metal can decelerate the melt flow and help humping mitigation.
(4) The humping can be effectively suppressed by adding a TIG arc behind the laser beam. Under the effect of the arc force and increased melt pool size, the high-speed melt flow decelerates in advance, so humping is avoided.

## Figures and Tables

**Table 1:** Welding parameters in the experiment.

No.	Laser Power (W)	Welding Speed (m/min)	Linear Energy Density (J/m)
1	2000	16	7500
2	3000	24	7500

**Table 2:** Parameters used in the simulation [21].

Property	Value
$\mathrm{Density}\;\mathrm{of}\;\mathrm{solid}\;\mathrm{metal},\;\rho_{s}$ (kg ∙ m^−3^)	7790
$\mathrm{Density}\;\mathrm{of}\;\mathrm{liquid}\;\mathrm{metal},\;\rho_{l}$ (kg ∙ m^−3^)	8280 − 0.8 *T*
$\mathrm{Thermal}\;\mathrm{conductivity}\;\mathrm{of}\;\mathrm{solid}\;\mathrm{metal},\;k_{s}$ (W ∙ m^−1^ ∙ K^−1^)	10.15 + 0.0152 *T*
$\mathrm{Thermal}\;\mathrm{conductivity}\;\mathrm{of}\;\mathrm{liquid}\;\mathrm{metal},\;k_{l}$ (W ∙ m^−1^ ∙ K^−1^)	5.03 + 0.0133 *T*
Dynamic viscosity, μ (kg ∙ m^−1^ ∙ s^−1^)	0.006
Specific heat, c_p_ (J ∙ kg^−1^ ∙ K^−1^)	780
$\mathrm{Latent}\;\mathrm{heat}\;\mathrm{of}\;\mathrm{fusion},\;L_{m}$ (J ∙ kg^−1^)	2.47 × 10^5^
$\mathrm{Latent}\;\mathrm{heat}\;\mathrm{of}\;\mathrm{vaporization},\;L_{v}$ (J ∙ kg^−1^)	6.34 × 10^6^
$\mathrm{Solidus}\;\mathrm{temperature},\;T_{s}$ (K)	1670
$\mathrm{Liquidus}\;\mathrm{temperature},\;T_{l}$ (K)	1727
$\mathrm{Boiling}\;\mathrm{temperature},\;T_{v}$ (K)	3200
Molar mass, *M* (kg ∙ mol^−1^)	0.056
$\mathrm{Convective}\;\mathrm{coefficient},\;h_{A}$ (W ∙ m^−2^ ∙ K^−1^)	20
$\mathrm{Emissivity},\;\varepsilon_{r}$	0.4
$\mathrm{Ambient}\;\mathrm{temperature},\;T_{\infty }$ (K)	300
$\mathrm{Ambient}\;\mathrm{pressure},\;P_{0}$ (Pa)	1.013 × 10^5^
$\mathrm{Small}\;\mathrm{constant},\;C_{K}$	0.001
$\mathrm{Mushy}\;\mathrm{zone}\;\mathrm{constant},\;C_{\mathrm{mush}}$	1 × 10^9^

**Table 3:** Comparison of the width and depth of the weld seam between experiments and simulations.

Welding Parameter	Depth			Width		
	Experiment (mm)	Simulation (mm)	Error (%)	Experiment (mm)	Simulation (mm)	Error (%)
2000 W, 16 m/min	0.565	0.545	3.5	0.536	0.572	6.2
3000 W, 24 m/min	0.574	0.597	4.0	0.540	0.565	4.6

**Table 4:** Positions of measuring cross sections and measurement results of flowrates.

		Swelling 2 (*t* = 15 ms)	Swelling 3 (*t* = 22 ms)	Swelling 4 (*t* = 24 ms)
*x* (mm)	A	6.5	9.3	10.1
	B	5.6	6.4	7.7
	C	4.6	5.4	6.7
*q* (mm^3^/s)	A	27.1	31.4	28.3
	B	20.8	13.3	18.5
	C	3.9	0.6	1.3
∆q/∆x (mm^2^/s)	AB	−7	−6.2	−4.1
	BC	−16.9	−12.7	−17.2

## Data Availability

The data presented in this study are available on request from the corresponding author.
